# Comparison of the effects of perioperative fentanyl and morphine use on the short-term prognosis of patients with cardiac surgery in the ICU

**DOI:** 10.3389/fphar.2024.1453835

**Published:** 2025-02-17

**Authors:** Jingtao Zhong, Runqian Sui, Jie Zi, Anbiao Wang

**Affiliations:** Department of Cardiac Surgery, Shandong Provincial Hospital Affiliated to Shandong First Medical University, Jinan, Shandong, China

**Keywords:** fentanyl, morphine, cardiac surgery, delirium, in-hospital mortality

## Abstract

**Aim:**

This study aimed to analyze the effects of perioperative morphine and fentanyl use on delirium, length of ICU stay, and in-hospital mortality after cardiac surgery.

**Methods:**

This cohort study retrieved the data of 4,596 patients admitted to ICU after cardiac surgery in MIMIC-IV database from 2008 to 2019. The primary outcome was postoperative delirium. The secondary outcomes were length of ICU stay, and in-hospital mortality. To compare the effects of perioperative fentanyl and morphine use on postoperative delirium, length of ICU stay, and in-hospital mortality, univariate, two-way stepwise, as well as multivariable Logistic regression, linear regression, and Cox proportional hazards models was respectively used. Odd ratio (OR), β coefficient, hazard ratio (HR), and respective confidence interval (CI) were calculated. Subgroup analysis was conducted in terms of age, use of extracorporeal circulation, midazolam, dexmedetomidine or surgery type.

**Results:**

In total, the data of 4,596 patients were analyzed including 2,589 morphine user and 2007 fentanyl user. We found that the risk of postoperative delirium was reduced in patients with cardiac surgery undergoing perioperative morphine relative to perioperative fentanyl (OR = 0.62, 95%CI: 0.40–0.96). Perioperative morphine use was negatively correlated with the length of ICU stay in patients with cardiac surgery in comparison to patients with perioperative fentanyl use (β = −0.72, 95%CI: −1.06, −0.39). Relative to patients who had perioperative fentanyl, patients who had perioperative morphine were associated with reduced risk of in-hospital mortality in patients with cardiac surgery after adjusting for respective confounding factors (HR = 0.35, 95%CI: 0.13–0.91).

**Conclusion:**

Perioperative morphine use was related to lower risk of delirium and in-hospital mortality as well as shortened length of ICU stay in these patients. The findings might offer a reference for perioperative anesthesia management in patients receiving cardiac surgery.

## Introduction

Cardiac surgery is one of the treatments of adult heart diseases, which can reduce the mortality of patients (Ye et al., 2022). With the increase of aging population, the incidence of cardiovascular disease (CVD) rise, which leads to the increased heart surgery (Pittams et al., 2022). Every year, there were about 2 million people undergoing cardiac surgery (Sousa-Uva et al., 2018). Coronary artery bypass grafting (CABG) is a major surgical procedure involving the creation of new pathways to bypass blockages caused by atheromatous plaques in the coronary arteries (McNichols et al., 2021). Surgical aortic valve replacement (SAVR) has been one of the treatments for heart disease and has been associated with improved survival rates and enhanced quality of life (Kalogeropoulos et al., 2022). Although the advancements in treatment methods have help reduce the mortality of patients, the significant incidence of postoperative complications and their impact on hospital stay duration and mortality rates remains crucial clinical challenges (Pittams et al., 2022; Ball et al., 2016). Therefore, the identification of factors that influence the prognosis of patients receiving cardiac surgery may be useful for risk stratification and individualized treatments.

Opioid analgesia is the cornerstone of anesthesia management during cardiac surgery, and opioid use is associated with poor outcomes such as postoperative delirium in patients (Bilotta et al., 2013; Sanson et al., 2018). Morphine and fentanyl are commonly used opioids in the perioperative period (Botea et al., 2023). Fentanyl is known to have a shorter onset of action and a more rapid peak of action, with better penetration into the central nervous system. In contrast, morphine is rapidly metabolized to metabolically active glucuronides (van Dorp et al., 2006). Both are opiates with respiratory adverse effects, but fentanyl is also more potent, requiring a concentration 10 timeless than that for morphine (Suarez et al., 2010). Morphine has immunomodulatory properties, and can selectively inhibit inflammation cell activation while fentanyl showed no downregulation of inflammatory cells function after surgery (Murphy et al., 2007; Murphy et al., 2009; Bencsics et al., 1997). Fentanyl does not bind to the μ3 receptor, and it does not appear to induce downregulation of inflammatory cell function in cellular models or in humans following surgical procedures (Taylor et al., 1997). Previous studies revealed that the effects of morphine and fentanyl on the complications of patients receiving different surgery were inconsistent (Murphy et al., 2007; Murphy et al., 2009; Casamento et al., 2023; Hu et al., 2021). Therefore, to compare the effects of fentanyl and morphine use on the short-term prognosis of patients with cardiac surgery in the ICU was necessary.

This study aims to analyze the effects of perioperative morphine and fentanyl use on delirium, length of ICU stay, and in-hospital mortality after cardiac surgery based on the data from the Medical Information Mart for Intensive Care-IV (MIMIC-IV) database. Subgroup analysis was also performed concerning age, use of extracorporeal circulation, midazolam, dexmedetomidine or surgery type.

## Methods

### Study design and population

This cohort study retrieved the data of 6,395 patients admitted to ICU after cardiac surgery in MIMIC-IV database from 2008 to 2019. The MIMIC-IV database is a relational database that encompasses authentic hospital admissions data from a prominent tertiary academic medical center in Boston, MA, United States, which provides comprehensive patient information during their hospitalization period, including laboratory measurements, administered medications, documented vital signs, and other relevant details (Johnson et al., 2023). Patients who underwent cardiac surgery were screened from MIMIC-IV according to the International Classification of Diseases (ICD) codes [CABG (ICD-9: 3,610–3,619, and ICD-10: first four digits were 0,210–0,213), valvular surgery (ICD-9: 3,599, 3,500–3,504, 3,510–3,514, and 3,520–3,528, and ICD-10: first four digits were 02QF, 02QG, 02QH, 02QJ, 02RF, 02RG, 02RH, and 02RJ), repair of septal defect of heart (ICD-9: 3,550–3,555, 3,560, 3,570, 3,571, and 3,598), aortic replacement (ICD-9: 3,845, and ICD-10: first four digits were 02RW, and 02RX)]. Participants without information of perioperative morphine and fentanyl use from MIMIC-IV database, those with the age <18 years old, and the length of ICU stay less than 24 h were excluded. The project was approved by the Institutional Review Boards of Beth Israel Deaconess Medical Center (Boston, MA) and the Massachusetts Institute of Technology (Cambridge, MA). Requirement for individual patient consent was waived from the Ethics Committee of Shandong Provincial Hospital Affiliated to Shandong First Medical University because the project did not impact clinical care and all protected health information was deidentified.

### Potential covariates and definitions

Age (years), gender (males or females), ethnicity (Black, White, others, or unknown), insurance (Medicaid, Medicare or others), marital status (married, non-married, unknown), 24 h urine output (mL), alcohol abuse or not, weight (kg), Simplified Acute Physiology Score II (SAPSII), Charlson comorbidity index, heart rate (beat/min), systolic blood pressure (mmHg), diastolic blood pressure (mmHg), central venous pressure (CVP) (mmHg), respiratory rate (beat/min), temperature (°C), oxygen saturation (SpO_2_) (%), white blood cell (WBC) (K/uL), platelet (K/uL), red cell distribution width (RDW) (%), hematocrit (%), international normalized ratio (INR), blood urea nitrogen (BUN) (mg/dL), glucose (mg/dL), calcium (mmol/L), sodium (mEq/L), potassium (mEq/L), chloride (mEq/L), bicarbonate (mEq/L), lactate (mmol/L), PH, arterial partial pressure of carbon dioxide (PaCO_2_) (mmHg), partial pressure of oxygen (PaO_2_) (mmHg), surgery type (aortic replacement, CABG, combined cardiac surgery, repair of septal defect of heart or valvular surgery), extracorporeal circulation or not, ventilation or not, midazolam or not, propofol or not, dexmedetomidine or not, antiplatelet or not, anticoagulation or not, estimated glomerular filtration rate (eGFR), or anemia or not.

Antiplatelet referred to tirofiban (itemID: 225157). Anticoagulation included Warfarin (itemID: 225913), heparin (itemID: 225152, 229597, and 225975), Bivalirudin (itemID: 225148, and 229781), argatroban (itemID: 225147), Lepirudin (itemID: 221892), and fondaparinux (itemID: 225908). With postoperative delirium as the outcome, the use of antiplatelet and anticoagulant therapy was evaluated from ICU admission to 24 h ICU admission. With the length of ICU stay, and in-hospital mortality as the outcomes, antiplatelet and anticoagulant drugs use was evaluated from ICU admission to ICU discharge. eGFRCKD-EPI (mL/min/1.73 m^2^) = 141 × min (Scr/κ, 1) α × max (Scr/κ, 1)-1.029 × 0.993 age × 1.108 (if female) × 1.159 (if black), κ is 0.7 for females and 0.9 for males, α is −0.329 for females and −0.411 for males, min indicates the minimum of Scr/κ or 1, and max indicates the maximum of Scr/κ or 1 (Levey et al., 2009).

### Main variables

Perioperative morphine and fentanyl use were main variables analyzed in this study. Perioperative morphine use was obtained according to itemID (225154), and perioperative fentanyl use was identified via itemID (221744, 225942, and 225972).

## Outcomes

The primary outcome was postoperative delirium. Postoperative delirium judgment according to the ICD-9 codes: ICD -281, 2930, 2931, 2939, 34831, 34982, 78009, 78097, ICD-10: F05, G92, G9341, R410, and R4182 (Chen et al., 2023). The secondary outcomes were length of ICU stay, and in-hospital mortality.

### Statistical analysis

The normality of quantitative data was tested by skewness and kurtosis methods, and the homogeneity of variance was tested by Levene’s test. Normally distributed measurement data were described as Mean and standard deviation [Mean (±SD)]. The t-test was used for comparison between groups with equal variance, and the t’ test was used for uneven variance. Non-normally distributed measurement data were displayed as the median and quarters [M (Q₁, Q₃)], and comparison between groups using Wilcoxon rank sum test. Enumeration data were described as the number and ratio of cases [n (%)], and chi-square test or Fisher’s exact test was used for comparison between groups. The missing values variables were shown in Supplementary Table S1. Random forest interpolation was applied for variables with missing values < 20%. To compare the effects of perioperative fentanyl and morphine use on postoperative delirium, univariate Logistic regression model was used to explore the confounding factors correlated with postoperative delirium. Variables with statistical difference (*P* < 0.05) in univariate Logistic regression model were subjected to two-way stepwise regression. Two-way stepwise regression model was conducted via removing variables that are not significant by t-test from the regression model, and introducing new variables that are significant by F-test into the regression model. *P* < 0.05 was regarded as statistical significance. To compare the effects of perioperative fentanyl and morphine use on length of ICU stay, univariate linear regression model and two-way stepwise regression model were applied. Univariate Cox proportional hazards models followed by two-way stepwise regression was employed to compare the effects of perioperative fentanyl and morphine use on in-hospital mortality. Subgroup analysis was conducted in terms of age, use of extracorporeal circulation, midazolam, dexmedetomidine or surgery type. Odd ratio (OR), β coefficient, hazard ratio (HR), and respective confidence interval (CI) were calculated. Sensitivity analysis, difference comparison, and statistical analysis were performed using R version 4.3.1 (2023–06-16 ucrt).

## Results

### Comparisons of characteristics of participants with perioperative fentanyl or morphine use

**FIGURE 1:**
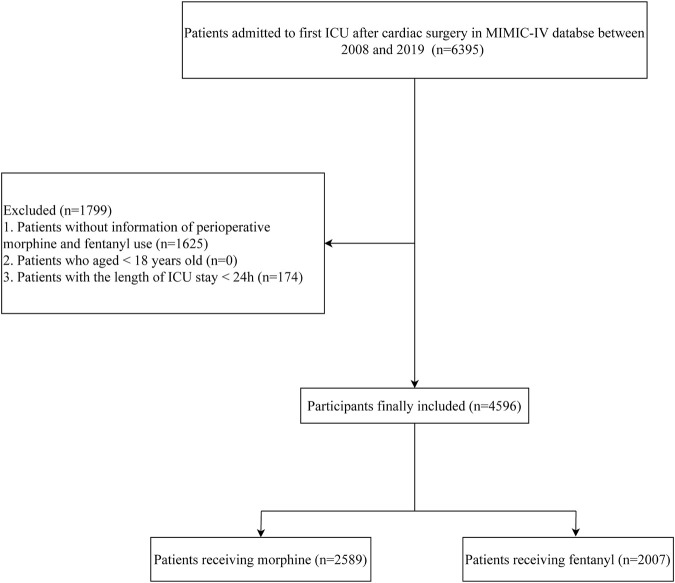
The screening process of the participants.

In total, 6,395 patients admitted to ICU after cardiac surgery were extracted from MIMIC-IV database. Among them, 1,625 patients without information of perioperative fentanyl or morphine use from MIMIC-IV database were excluded. Also, those stayed in ICU less than 24 h were not included (n = 174). Finally, the data of 4,596 patients were analyzed including 2,589 morphine user and 2007 fentanyl user. The flow chart of participants was shown in Figure 1.

The mean age of participants with perioperative morphine use was older than those receiving perioperative fentanyl (67.92 years vs. 66.99 years). The percentages of female patients with perioperative morphine use were higher than those receiving perioperative fentanyl (31.09% vs. 26.66%). Significant difference was observed among the percentage of patients with ethnicities, marital status (*P* < 0.05). The 24 h urine output in participants with perioperative morphine use was higher than those receiving perioperative fentanyl (2085.79 mL vs. 1787.16 mL). The Charlson comorbidity index in partients with perioperative morphine use was lower than those receiving perioperative fentanyl (1.78 vs. 2.10). The percentages of patients receiving different surgery types were statistical different between perioperative morphine use group and perioperative fentanyl use group. The percentage of patients receiving extracorporeal circulation in the perioperative morphine use group was higher than the perioperative fentanyl use group (96.37% vs. 8.17%). The percentage of patients with delirium in the perioperative morphine use group was lower than the perioperative fentanyl use group (5.41% vs. 11.16%). The mean length of ICU stay in the perioperative morphine use group was lower than the perioperative fentanyl use group (1.48 days vs. 1.78 days). The mortality rate in patients with perioperative morphine use was higher than patients with perioperative fentanyl use (1.44% vs. 0.27%). More detailed information on the characteristics of participants with perioperative fentanyl or morphine use was presented in Table 1.

**TABLE 1:** The baseline characteristics of patients receiving fentanyl or morphine.

Variables	Total (n = 4,596)	Fentanyl (n = 2007)	Morphine (n = 2,589)	Statistics	*P*
Age, years, Mean (±SD)	67.51 (±10.86)	66.99 (±10.45)	67.92 (±11.15)	t' = −2.902	0.004
Gender, n (%)				χ^2^ = 10.559	0.001
Female	1,340 (29.16)	535 (26.66)	805 (31.09)		
Male	3,256 (70.84)	1,472 (73.34)	1784 (68.91)		
Ethnicity, n (%)				χ^2^ = 84.064	<0.001
Black	172 (3.74)	86 (4.29)	86 (3.32)		
Others	565 (12.29)	221 (11.01)	344 (13.29)		
Unknown	493 (10.73)	307 (15.3)	186 (7.18)		
White	3,366 (73.24)	1,393 (69.41)	1973 (76.21)		
Insurance, n (%)				χ^2^ = 3.666	0.160
Medicaid	177 (3.85)	65 (3.24)	112 (4.33)		
Medicare	1989 (43.28)	878 (43.75)	1,111 (42.91)		
Others	2,430 (52.87)	1,064 (53.01)	1,366 (52.76)		
Marital status, n (%)				χ^2^ = 89.273	<0.001
Married	2,847 (61.95)	1,195 (59.54)	1,652 (63.81)		
Non-married	1,473 (32.05)	616 (30.69)	857 (33.1)		
Unknown	276 (6.01)	196 (9.77)	80 (3.09)		
24 h urine output, mL, Mean (±SD)	1955.38 (±878.58)	1787.16 (±814.95)	2085.79 (±903.75)	t' = −11.746	<0.001
Alcohol abuse, n (%)				χ^2^ = 0.281	0.596
No	4,542 (98.83)	1981 (98.7)	2,561 (98.92)		
Yes	54 (1.17)	26 (1.3)	28 (1.08)		
Weight, kg, M (Q₁, Q₃)	83.23 (71.85–96.32)	83.82 (72.56–97.3)	82.64 (71.17–95.12)	W = 2727651.5	0.004
SAPSII, score, Mean (±SD)	36.89 (±11.95)	36.86 (±11.56)	36.92 (±12.24)	t' = −0.161	0.872
Charlson comorbidity index, score, Mean (±SD)	1.92 (±1.72)	2.10 (±1.80)	1.78 (±1.64)	t' = 6.130	<0.001
Heart rate, bpm, Mean (±SD)	80.15 (±9.91)	79.40 (±10.07)	80.73 (±9.74)	t = −4.523	<0.001
Systolic blood pressure, mmHg, Mean (±SD)	111.94 (±16.52)	111.58 (±16.41)	112.23 (±16.60)	t = −1.316	0.188
Diastolic blood pressure, mmHg, Mean (±SD)	58.46 (±10.18)	57.89 (±9.79)	58.90 (±10.45)	t' = −3.364	0.001
CVP, mmHg, M (Q₁, Q₃)	10 (8–13)	9 (7–12)	11 (8.7–14)	W = 2033302	<0.001
Respiratory rate, M (Q₁, Q₃)	15 (14–16)	15.73 (14–16)	14 (13.78–16)	W = 3460784.5	<0.001
Temperature, Mean (±SD)	36.17 (±0.60)	36.21 (±0.59)	36.14 (±0.60)	t = 4.222	<0.001
SpO_2_, M (Q₁, Q₃)	100 (100–100)	100 (99–100)	100 (100–100)	W = 2458322.5	<0.001
WBC, K/uL, M (Q₁, Q₃)	12.5 (9.5–16)	12.7 (9.8–16.1)	12.4 (9.3–15.9)	W = 2687265.5	0.046
Platelet, K/uL, Mean (±SD)	149.89 (±52.04)	139.76 (±47.85)	157.74 (±53.79)	t' = −11.962	<0.001
Hemoglobin, g/dL, Mean (±SD)	9.86 (±2.10)	10.11 (±2.12)	9.68 (±2.06)	t = 6.912	<0.001
RDW, %, Mean (±SD)	13.77 (±1.39)	13.62 (±1.49)	13.89 (±1.29)	t' = −6.467	<0.001
Hematocrit, %, Mean (±SD)	29.59 (±6.28)	30.37 (±6.37)	28.98 (±6.14)	t = 7.495	<0.001
Creatinine, mg/dL, M (Q₁, Q₃)	0.8 (0.7–1)	0.9 (0.7–1.1)	0.8 (0.7–1)	W = 2794942	<0.001
INR, ratio, M (Q₁, Q₃)	1.4 (1.3–1.5)	1.4 (1.3–1.6)	1.4 (1.3–1.5)	W = 2977516.5	<0.001
BUN, mg/dL, M (Q₁, Q₃)	16 (13–20)	15 (12.5–19)	16 (13–20)	W = 2450139.5	0.001
Glucose, mg/dL, Mean (±SD)	141.61 (±38.76)	145.30 (±42.13)	138.76 (±35.68)	t' = 5.573	<0.001
Calcium, mmol/L, M (Q₁, Q₃)	1.17 (1.1–1.27)	1.14 (1.08–1.21)	1.2 (1.12–1.3)	W = 1869794	<0.001
Sodium, mEq/L, Mean (±SD)	135.64 (±2.81)	135.20 (±2.88)	135.97 (±2.70)	t' = −9.156	<0.001
Potassium, mEq/L, Mean (±SD)	4.68 (±0.76)	4.59 (±0.72)	4.74 (±0.78)	t' = −7.032	<0.001
Chloride, mEq/L, M (Q₁, Q₃)	107 (105–109)	107 (105–109)	107 (104–109)	W = 2437624.5	<0.001
Bicarbonate, mEq/L, Mean (±SD)	23.03 (±2.23)	22.63 (±2.15)	23.35 (±2.24)	t = −11.096	<0.001
Lactate, mmol/L, Mean (±SD)	2.29 (±1.06)	2.26 (±1.14)	2.31 (±1.00)	t = −1.622	0.105
PH, Mean (±SD)	7.40 (±0.06)	7.40 (±0.06)	7.41 (±0.06)	t' = −5.797	<0.001
PaCO_2_, mmHg, Mean (±SD)	41.06 (±6.51)	41.19 (±6.38)	40.95 (±6.62)	t' = 1.239	0.215
PaO_2_, mmHg, Mean (±SD)	319.17 (±99.04)	296.72 (±101.44)	336.57 (±93.53)	t' = −13.663	<0.001
Surgery type, n (%)				χ^2^ = 552.653	<0.001
Aortic replacement	162 (3.52)	122 (6.08)	40 (1.54)		
CABG	2,905 (63.21)	1,580 (78.72)	1,325 (51.18)		
Combined cardiac surgery	709 (15.43)	144 (7.17)	565 (21.82)		
Repair of septal defect of heart	16 (0.35)	1 (0.05)	15 (0.58)		
Valvular surgery	804 (17.49)	160 (7.97)	644 (24.87)		
Extracorporeal circulation, n (%)				χ^2^ = 3,603.229	<0.001
No	1937 (42.15)	1843 (91.83)	94 (3.63)		
Yes	2,659 (57.85)	164 (8.17)	2,495 (96.37)		
Ventilation, n (%)				χ^2^ = 0.000	1.000
No	52 (1.13)	23 (1.15)	29 (1.12)		
Yes	4,544 (98.87)	1984 (98.85)	2,560 (98.88)		
Midazolam, n (%)				χ^2^ = 4.368	0.037
No	4,426 (96.3)	1919 (95.62)	2,507 (96.83)		
Yes	170 (3.7)	88 (4.38)	82 (3.17)		
Propofol, n (%)				χ^2^ = 19.921	<0.001
No	57 (1.24)	42 (2.09)	15 (0.58)		
Yes	4,539 (98.76)	1965 (97.91)	2,574 (99.42)		
Dexmedetomidine, n (%)				χ^2^ = 37.546	<0.001
No	3,575 (77.79)	1,475 (73.49)	2,100 (81.11)		
Yes	1,021 (22.21)	532 (26.51)	489 (18.89)		
Antiplatelet, n (%)				χ^2^ = 3.795	0.051
No	19 (0.41)	13 (0.65)	6 (0.23)		
Yes	4,577 (99.59)	1994 (99.35)	2,583 (99.77)		
Anticoagulation, n (%)				χ^2^ = 0.809	0.368
No	2,559 (55.68)	1,133 (56.45)	1,426 (55.08)		
Yes	2037 (44.32)	874 (43.55)	1,163 (44.92)		
eGFR, mL/min/1.73m^2^, M (Q₁, Q₃)	102.35 (93.62–116.49)	103.1 (93.82–118.59)	101.61 (93.16–115.13)	W = 2754321	<0.001
Anemia, n (%)				χ^2^ = 12.544	<0.001
No	4,132 (89.9)	1768 (88.09)	2,364 (91.31)		
Yes	464 (10.1)	239 (11.91)	225 (8.69)		
Delirium, n (%)				χ^2^ = 50.532	<0.001
No	4,232 (92.08)	1783 (88.84)	2,449 (94.59)		
Yes	364 (7.92)	224 (11.16)	140 (5.41)		
Length of ICU stay, days, M (Q₁, Q₃)	1.54 (1.24–3.05)	1.78 (1.25–3.15)	1.48 (1.23–3)	W = 2756292.5	<0.001
In-hospital follow time, days, M (Q₁, Q₃)	5.22 (4.26–7.06)	5.27 (4.3–7.2)	5.18 (4.23–6.31)	W = 2888455.5	<0.001
Hospital expire flag, n (%)				χ^2^ = 18.587	<0.001
Survival	4,560 (99.22)	1978 (98.56)	2,582 (99.73)		
Death	36 (0.78)	29 (1.44)	7 (0.27)		

SD, standard deviation; M, median; Q₁, 1st Quartile; Q₃, 3rd Quartile; t, Student's t test; t', Satterthwaite t-test; W, wilcoxon rank sum test; χ^2^, Chi-square test; SAPSII, Simplified Acute Physiology Score II; CVP, central venous pressure; SpO_2_, oxygen saturation; RDW, red cell distribution width; INR, international normalized ratio; BUN, blood urea nitrogen; PaO_2_, partial pressure of oxygen; CABG, coronary artery bypass graft; eGFR, estimated glomerular filtration rate.

The distributions of perioperative fentanyl dose or perioperative morphine dose were exhibited in Supplementary Figure S1. Most of the patients who received fentanyl received doses less than 5 mg, while Most of the patients who received fentanyl received doses less than 20 mg.

### Comparisons of the effects of perioperative fentanyl use and perioperative morphine use on postoperative delirium in patients with cardiac surgery

The screen process of confounding factors associated with postoperative delirium in patients with cardiac surgery were exhibited in Supplementary Table S2. The results revealed that age, marital status, Charlson comorbidity index, SpO_2_, RDW, hematocrit, potassium, bicarbonate, surgery type, extracorporeal circulation, midazolam, dexmedetomidine, and eGFR were confounding factors. Compared to patients receiving perioperative fentanyl, those receiving perioperative morphine might be associated with decreased risk of postoperative delirium in patients with cardiac surgery (OR = 0.46, 95%CI: 0.37–0.57). After confounding factors were adjusted, the risk of postoperative delirium was reduced in patients with cardiac surgery undergoing perioperative morphine relative to perioperative fentanyl (OR = 0.62, 95%CI: 0.40–0.96) (Table 2).

**TABLE 2:** The effects of perioperative fentanyl use and perioperative morphine use on postoperative delirium in patients with cardiac surgery.

	Model 1		Model 2	
Variables	OR (95% CI)	*P*	OR (95% CI)	*P*
Drug type				
Fentanyl	Ref		Ref	
Morphine	0.46 (0.37–0.57)	<0.001	0.62 (0.40–0.96)	0.032

OR, odds ratio; CI, confidence intervals; Ref, reference.

Model 1: the crude model.

Model 2: Adjusted for age, marital status, Charlson comorbidity index, SpO2, RDW, hematocrit, potassium, bicarbonate, surgery type, extracorporeal circulation, midazolam, dexmedetomidine, and eGFR.

### Comparisons of the effects of perioperative fentanyl use and perioperative morphine use on length of ICU stay in patients with cardiac surgery

Age, gender, 24 h urine output, SAPSII, Charlson comorbidity index, heart rate, systolic blood pressure, SpO_2_, RDW, INR, glucose, bicarbonate, lactate, surgery type, extracorporeal circulation, midazolam, dexmedetomidine, anticoagulation, and delirium were confounders related to length of ICU stay in patients with cardiac surgery (Supplementary Table S3). In the adjusted group, perioperative morphine use was negatively correlated with the length of ICU stay in patients with cardiac surgery in comparison to patients with perioperative fentanyl use (β = −0.72, 95%CI: −1.06, −0.39) (Table 3).

**TABLE 3:** The effects of perioperative fentanyl use and perioperative morphine use on length of ICU stay in patients with cardiac surgery.

	Model 1		Model 2	
Variables	β (95% CI)	*P*	β (95% CI)	*P*
Drug type				
Fentanyl	Ref		Ref	
Morphine	−0.62 (−0.80, −0.45)	<0.001	−0.72 (−1.06, −0.39)	<0.001

β, Coefficient; CI, confidence intervals; Ref, reference.

Model 1: the crude model.

Model 2: adjusted for age, gender, 24 h urine output, SAPSII, charlson comorbidity index, heart rate, systolic blood pressure, SpO2, RDW, INR, glucose, bicarbonate, lactate, surgery type, extracorporeal circulation, midazolam, dexmedetomidine, anticoagulation, and delirium.

### Comparisons of the effects of perioperative fentanyl use and perioperative morphine use on in-hospital mortality in patients with cardiac surgery

The results in Supplementary Table S4 indicated that SAPSII, CVP, calcium, bicarbonate, lactate, surgery type, midazolam, antiplatelet, delirium, and length of ICU stay were confounding factors related to in-hospital mortality in patients with cardiac surgery. Relative to patients who received perioperative fentanyl, patients who received perioperative morphine were associated with reduced risk of in-hospital mortality after adjusting for respective confounding factors (HR = 0.35, 95% CI: 0.13–0.91) (Table 4).

**TABLE 4:** The effects of perioperative fentanyl use and perioperative morphine use on in-hospital mortality in patients with cardiac surgery.

	Model 1		Model 2	
Variables	HR (95% CI)	*P*	HR (95% CI)	*P*
Drug type				
Fentanyl	Ref		Ref	
Morphine	0.25 (0.11–0.58)	0.001	0.35 (0.13–0.91)	0.032

HR, hazard ratio; CI, confidence intervals; Ref, reference.

Model 1: the crude model.

Model 2: adjusted for SAPSII, CVP, calcium, bicarbonate, lactate, surgery type, midazolam, antiplatelet, delirium, and length of ICU, stay.

### Subgroup analysis of the effects of perioperative fentanyl use and perioperative morphine use on postoperative delirium or length of ICU stay in patients with cardiac surgery

**FIGURE 2:**
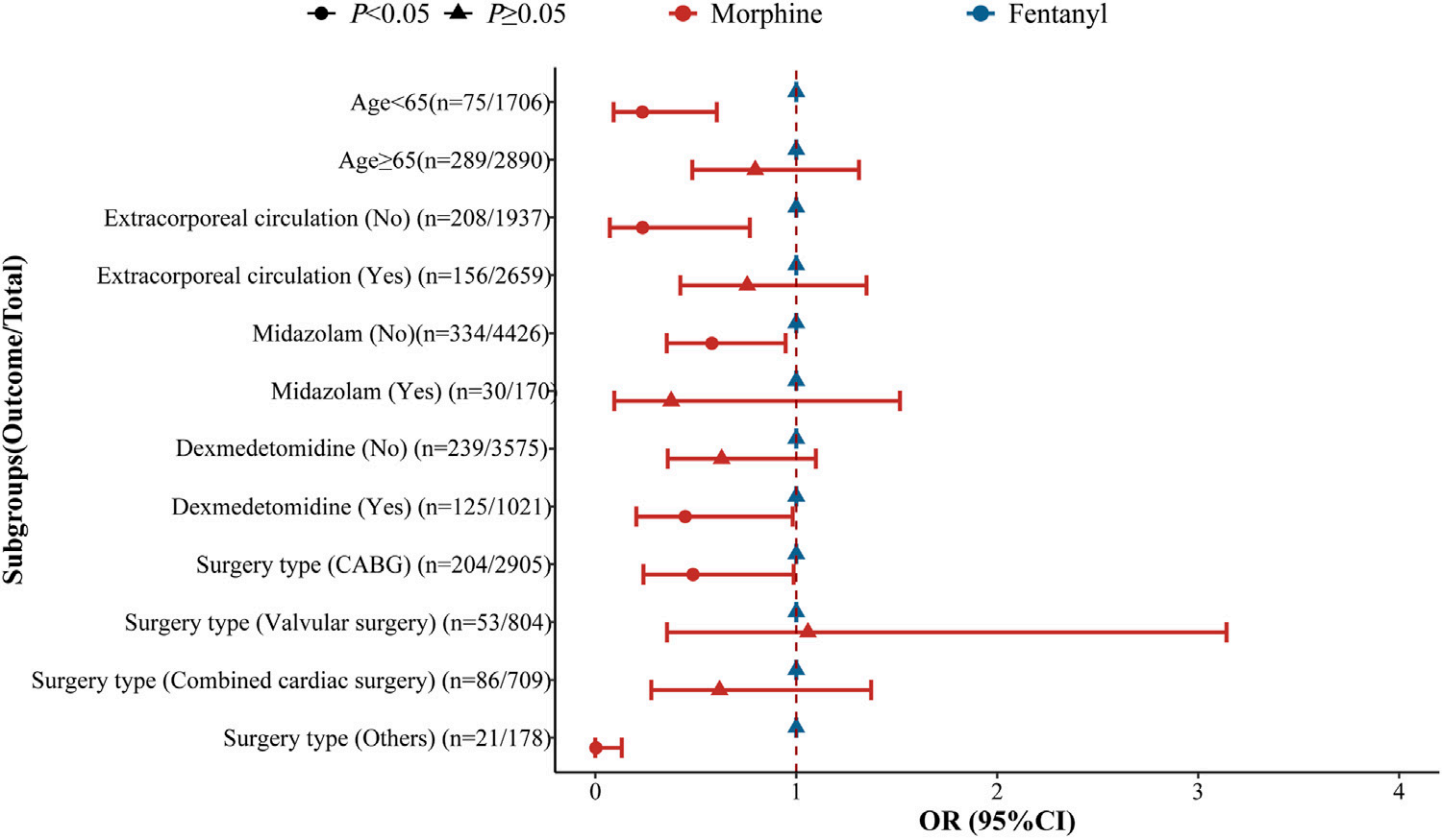
Subgroup analysis of the effects of perioperative fentanyl use and perioperative morphine use on postoperative delirium in patients with cardiac surgery.

**FIGURE 3:**
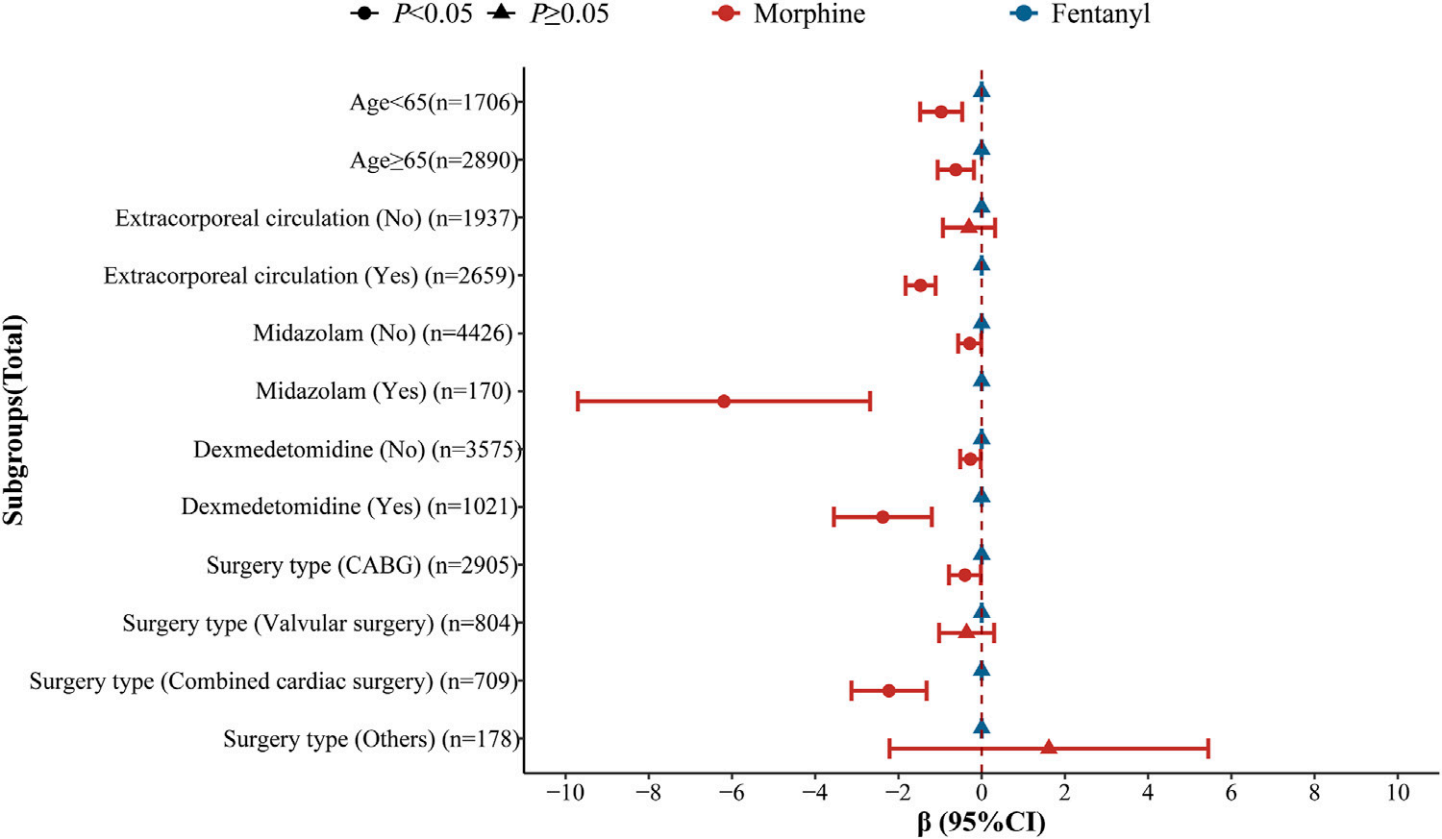
Subgroup analysis of the effects of perioperative fentanyl use and perioperative morphine use on length of ICU stay in patients with cardiac surgery.

As shown in Figure 2, the risk of postoperative delirium was reduced in the perioperative morphine group compared to perioperative fentanyl group among those <65 years old. For patients who did not use extracorporeal circulation, the risk of postoperative delirium was reduced in the perioperative morphine group. For patients not using midazolam, the risk of postoperative delirium was decreased in the perioperative morphine group. The decreased risk of postoperative delirium was observed and those who used dexmedetomidine and those receiving other type of surgery in the perioperative morphine group. Perioperative morphine use was related to shortened length of ICU stay regardless the age. Perioperative morphine use was negatively correlated with decreased length of ICU stay compared with the perioperative fentanyl group in the population using extracorporeal circulation. In patients receiving midazolam, perioperative morphine was associated with decreased length of ICU stay. Also in patients receiving dexmedetomidine, the reduced length of ICU stay was identified in perioperative morphine group. In patients receiving combined cardiac surgery, perioperative morphine group was correlated with decreased length of ICU stay (Figure 3).

## Discussion

The present study compared the effects of perioperative morphine and fentanyl use on the risk of delirium, length of ICU stay, and in patients after cardiac surgery. The results delineated that the risk of postoperative delirium, and in-hospital mortality was reduced in patients with cardiac surgery undergoing perioperative morphine relative to perioperative fentanyl. The length of ICU stay was negatively related to perioperative morphine use compared to perioperative fentanyl use in patients receiving cardiac surgery. The findings might provide a reference for perioperative anesthesia management and disease burden reduction in patients undergoing cardiac surgery.

In a prior study, in patients undergoing elective cardiac surgery with cardiopulmonary bypass, the administration of 40 mg intraoperative morphine as part of a comprehensive anesthetic technique resulted in enhanced postoperative quality-of-life measures and improved pain control during recovery compared to fentanyl (Murphy et al., 2009). The effectiveness of low-dose continuous infusion of morphine exceeds that of fentanyl, resulting in a decreased requirement for rescue analgesics in the management of postoperative pain (Venkatraman et al., 2021). The effectiveness of patient-controlled analgesia with morphine in reducing post-uterine artery embolization pain surpasses that of patient-controlled analgesia with fentanyl (Kim et al., 2008). The results of a double-blind randomized controlled trial demonstrated that patients undergoing elective CABG with cardiopulmonary bypass who received morphine experienced a lesser increase in postoperative inflammatory markers and a lower incidence of postoperative hyperthermia compared to those who received fentanyl (Murphy et al., 2007). The use of fentanyl for analgosedation in mechanically ventilated patients is associated with a higher incidence of hospital inpatient delirium compared to morphine (Casamento et al., 2023). In the acute respiratory distress syndrome (ARDS) or risk in ICU patients with ARDS, fentanyl users of hospital mortality risk significantly lower than that of morphine users (Hu et al., 2021). These findings were allied with the results in our study. We identified that perioperative morphine use was associated with decreased postoperative delirium, and in-hospital mortality risk and reduced length of ICU in patients with cardiac surgery relative to perioperative fentanyl use. The possible reasons might be that the administration of morphine was associated with a significantly reduced release of inflammatory cytokines, a more pronounced inhibition of adhesion molecule expression, and a lower incidence of postoperative hyperthermia compared to the administration of fentanyl (Murphy et al., 2007). Furthermore, fentanyl and morphine differ in terms of the signal transduction mechanism underlying the antin-ociceptive effects. Additionally, fentanyl and morphine exhibit distinct signal transduction mechanisms underlying their antinociceptive effects (Morgan et al., 2020) as well as the induction of immunosuppression in animal models (Molina-Martínez et al., 2014). Subgroup analysis revealed that the use of extracorporeal circulation or midazolam in patients receiving cardiac surgery should be carefully evaluated as the medications behave differently based on other medications present (Kuntz et al., 2021).

Morphine possesses unique properties that may confer advantages for patients undergoing cardiac surgery. In animal studies, for instance, the administration of morphine before myocardial ischemia has been shown to reduce the size of myocardial infarction (Frässdorf et al., 2005). Morphine can downregulate immune and inflammatory responses via the μ3 receptor located on monocytes and granulocytes (Welters et al., 2000). Pretreatment of activated granulocytes and macrophages with morphine attenuated cytokine production and expression of adhesion molecules (Bencsics et al., 1997) and morphine administration diminished activation of these immune cells in a pig model of cardiopulmonary bypass (Bilfinger et al., 1996). Fentanyl neither binds to the μ3 receptor nor downregulates inflammatory cell function (Bilfinger et al., 1998).

Also, there were findings different from our results. Evidence reported that there was a statistically significant difference in ventilator-free days at Day 28 in mechanically ventilated ICU patients when fentanyl was used for analgosedation compared with morphine. Among adult patients requiring mechanical ventilation, compared with morphine, fentanyl infusion significantly increased the median number of ventilator-free days at Day 28 (Casamento et al., 2021). In a study conducted by Russo et al., the administration of fentanyl through continuous intravenous infusion was found to be associated with a reduced requirement for rescue analgesic medication, accelerated bowel recovery, and decreased length of hospital stay (Russo et al., 2017). The analgesic effects of morphine and fentanyl in out-of-hospital settings are comparable, although a higher dosage of fentanyl was required to achieve this outcome (Fleischman et al., 2010). Decreased gastrointestinal motility occurred significantly less frequently in the fentanyl group (Saarenmaa et al., 1999). This might due to the population difference or the dose difference. Also, there was unexplained heterogeneity of treatment effect according to age, which may be attributed to variations in the pharmacokinetics of fentanyl, characterized by a significantly longer terminal elimination half-life and reduced clearance in elderly individuals (Venkatraman et al., 2021; Bentley et al., 1982).

The study evaluated the effects of perioperative fentanyl and morphine use on the short-term prognosis of patients with cardiac surgery based on MIMIC-IV database with a large sample size, which might provide reference for the analgesic management of patients undergoing cardiac surgery. Successful intervention programs including all aspects of good medical and nursing care in patients receiving cardiac surgery should be evaluated. However, the MIMIC-IV is a single center study, which might have some selection bias. Due to the low hospital mortality rate, subgroup analysis was not performed. Limited by database records such as preoperative health status and lack of access to relevant data outside the hospital, the long-term effects of morphine and fentanyl on patients undergoing cardiac surgery need to be further studied.

## Conclusion

The effects of perioperative fentanyl and morphine use on the delirium, in-hospital mortality or length of ICU stay of patients with cardiac surgery were compared in the current study. We found that perioperative morphine use was associated with lower risk of delirium and in-hospital mortality as well as shortened length of ICU stay in these patients. The findings might offer a reference for perioperative anesthesia management in patients receiving cardiac surgery.

## Data Availability

Publicly available datasets were analyzed in this study. This data can be found here: MIMIC-IV database, https://mimic.physionet.org/iv/.

## Supplementary material

SUPPLEMENTARY FIGURE S1 The doses of perioperative fentanyl use and perioperative morphine use in patients.
