# Supplementary material for: Comparison of the effects of perioperative fentanyl and morphine use on the short-term prognosis of patients with cardiac surgery in the ICU
Source: Front Pharmacol. 2025 Feb 17;15:1453835. doi: 10.3389/fphar.2024.1453835 (PMC11873745; doi:10.3389/fphar.2024.1453835)
Supplement: Supplementary file 2 [file Table1.docx]

Supplementary Table 1 The status of missing values of variables

|  | n | % | Imputation |
| --- | --- | --- | --- |
| Diastolic blood pressure | 1 | 0.02% | Random forest interpolation |
| Systolic blood pressure | 1 | 0.02% | Random forest interpolation |
| Heart rate | 2 | 0.04% | Random forest interpolation |
| WBC | 4 | 0.09% | Random forest interpolation |
| Hemoglobin | 4 | 0.09% | Random forest interpolation |
| INR | 4 | 0.09% | Random forest interpolation |
| Hematocrit | 4 | 0.09% | Random forest interpolation |
| PH | 4 | 0.09% | Random forest interpolation |
| Bicarbonate | 4 | 0.09% | Random forest interpolation |
| Chloride | 4 | 0.09% | Random forest interpolation |
| Potassium | 4 | 0.09% | Random forest interpolation |
| RDW | 4 | 0.09% | Random forest interpolation |
| Glucose | 4 | 0.09% | Random forest interpolation |
| BUN | 4 | 0.09% | Random forest interpolation |
| Sodium | 4 | 0.09% | Random forest interpolation |
| Creatinine | 4 | 0.09% | Random forest interpolation |
| Platelet | 4 | 0.09% | Random forest interpolation |
| Calcium | 5 | 0.11% | Random forest interpolation |
| Weight | 40 | 0.87% | Random forest interpolation |
| CVP | 86 | 1.87% | Random forest interpolation |
| Lactate | 115 | 2.50% | Random forest interpolation |
| Respiratory rate | 740 | 16.10% | Random forest interpolation |
| Temperature | 802 | 17.45% | Random forest interpolation |
| Neutrophils | 1832 | 39.86% | Deletion |
| Lymphocytes | 1833 | 39.88% | Deletion |
| Total bilirubin | 4240 | 92.25% | Deletion |
| Height | 4369 | 95.06% | Deletion |
| Serum albumin | 4421 | 96.19% | Deletion |
| CK-MB | 4510 | 98.13% | Deletion |
| NT-probnp | 4595 | 99.98% | Deletion |

CVP: central venous pressure; RDW: red cell distribution width; INR: international normalized ratio; BUN: blood urea nitrogen

Supplementary Table 2 The screening process of the potential confounding factors associated with postoperative delirium

|  | Model 1 |  | Model 2 |  |
| --- | --- | --- | --- | --- |
| Variables | OR (95% CI) | *P* | OR (95% CI) | *P* |
| Age | 1.05 (1.04-1.06) | <0.001 | 1.05 (1.04-1.06) | <0.001 |
| Gender |  |  |  |  |
| Female | Ref |  |  |  |
| Male | 0.78 (0.62-0.98) | 0.032 |  |  |
| Ethnicity |  |  |  |  |
| Black | Ref |  |  |  |
| Others | 0.59 (0.34-1.05) | 0.071 |  |  |
| Unknown | 0.93 (0.54-1.61) | 0.809 |  |  |
| White | 0.61 (0.37-0.98) | 0.043 |  |  |
| Insurance |  |  |  |  |
| Medicaid | Ref |  |  |  |
| Medicare | 1.25 (0.72-2.16) | 0.429 |  |  |
| Others | 0.68 (0.39-1.18) | 0.166 |  |  |
| Marital status |  |  |  |  |
| Married | Ref |  | Ref |  |
| Non-married | 1.68 (1.34-2.10) | <0.001 | 1.52 (1.20-1.92) | 0.001 |
| Unknown | 1.71 (1.13-2.58) | 0.011 | 1.14 (0.73-1.76) | 0.564 |
| 24 h urine output | 1.00 (1.00-1.00) | <0.001 |  |  |
| Alcohol abuse |  |  |  |  |
| No | Ref |  |  |  |
| Yes | 1.19 (0.47-3.00) | 0.714 |  |  |
| Weight | 0.99 (0.98-1.00) | 0.001 |  |  |
| SAPSII | 1.03 (1.02-1.04) | <0.001 |  |  |
| Charlson comorbidity index | 1.22 (1.15-1.29) | <0.001 | 1.09 (1.02-1.16) | 0.011 |
| Heart rate | 1.01 (1.00-1.02) | 0.050 |  |  |
| Systolic blood pressure | 1.00 (0.99-1.00) | 0.565 |  |  |
| Diastolic blood pressure | 0.97 (0.96-0.98) | <0.001 |  |  |
| CVP | 1.00 (0.99-1.01) | 0.573 |  |  |
| Respiratory rate | 1.05 (1.02-1.08) | 0.002 |  |  |
| Temperature | 0.81 (0.68-0.97) | 0.020 |  |  |
| SPO2 | 0.94 (0.91-0.98) | 0.002 | 0.96 (0.92-1.00) | 0.057 |
| WBC | 1.00 (0.99-1.02) | 0.788 |  |  |
| Platelet | 1.00 (1.00-1.00) | 0.021 |  |  |
| RDW | 1.19 (1.12-1.27) | <0.001 | 1.06 (0.98-1.15) | 0.121 |
| Hematocrit | 0.96 (0.94-0.98) | <0.001 | 0.98 (0.96-1.00) | 0.032 |
| INR | 2.15 (1.51-3.05) | <0.001 |  |  |
| BUN | 1.02 (1.01-1.03) | 0.002 |  |  |
| Glucose | 1.00 (1.00-1.00) | 0.266 |  |  |
| Calcium | 1.04 (0.90-1.21) | 0.599 |  |  |
| Sodium | 0.96 (0.93-1.00) | 0.043 |  |  |
| Potassium | 0.84 (0.72-0.97) | 0.016 | 0.81 (0.69-0.95) | 0.009 |
| Chloride | 1.00 (0.99-1.02) | 0.592 |  |  |
| Bicarbonate | 0.86 (0.82-0.91) | <0.001 | 0.96 (0.91-1.01) | 0.081 |
| Lactate | 1.20 (1.10-1.31) | <0.001 |  |  |
| PH | 3.18 (0.57-17.58) | 0.185 |  |  |
| Paco2 | 0.98 (0.97-1.00) | 0.068 |  |  |
| Pao2 | 1.00 (1.00-1.00) | 0.524 |  |  |
| Surgery type |  |  |  |  |
| Aortic replacement | Ref |  | Ref |  |
| CABG | 0.51 (0.31-0.82) | 0.006 | 0.57 (0.34-0.95) | 0.031 |
| Combined cardiac surgery | 0.93 (0.56-1.54) | 0.771 | 1.10 (0.63-1.94) | 0.737 |
| Repair of septal defect of heart | 0.00 (0.00-Inf) | 0.970 | 0.00 (0.00-Inf) | 0.969 |
| Valvular surgery | 0.47 (0.28-0.81) | 0.006 | 0.65 (0.36-1.17) | 0.153 |
| Extracorporeal circulation |  |  |  |  |
| No | Ref |  | Ref |  |
| Yes | 0.52 (0.42-0.64) | <0.001 | 0.42 (0.32-0.55) | <0.001 |
| Ventilation |  |  |  |  |
| No | Ref |  |  |  |
| Yes | 1.41 (0.44-4.54) | 0.565 |  |  |
| Midazolam |  |  |  |  |
| No | Ref |  | Ref |  |
| Yes | 2.63 (1.74-3.96) | <0.001 | 1.89 (1.20-2.98) | 0.006 |
| Propofol |  |  |  |  |
| No | Ref |  |  |  |
| Yes | 1.56 (0.48-5.00) | 0.458 |  |  |
| Dexmedetomidine |  |  |  |  |
| No | Ref |  | Ref |  |
| Yes | 1.95 (1.55-2.45) | <0.001 | 1.98 (1.54-2.53) | <0.001 |
| Anticoagulation |  |  |  |  |
| No | Ref |  | Ref |  |
| Yes | 1.25 (0.99-1.57) | 0.056 | 1.82 (0.90-3.70) | 0.097 |
| EGFR | 1.00 (1.00-1.00) | 0.006 | 1.00 (1.00-1.00) | 0.020 |
| Anemia |  |  |  |  |
| No | Ref |  |  |  |
| Yes | 0.70 (0.46-1.04) | 0.079 |  |  |

OR: Odds ratio; CI: Confidence intervals; Ref: reference; SAPSII: Simplified Acute Physiology Score II; CVP: central venous pressure; SpO2: oxygen saturation; WBC: white blood cell; RDW: red cell distribution width; INR: international normalized ratio; BUN: blood urea nitrogen; PaO2: partial pressure of oxygen; CABG: coronary artery bypass graft; eGFR: estimated glomerular filtration rate

Model 1: the crude model

Model 2: multivariable model after bidirectional stepwise regression

Supplementary Table 3 The screening process of the potential confounding factors associated with length of ICU stay

|  | Model 1 |  | Model 2 |  |
| --- | --- | --- | --- | --- |
| Variables | β (95% CI) | *P* | β (95% CI) | *P* |
| Age | 0.03 (0.02, 0.03) | <0.001 | 0.01 (-0.00, 0.01) | 0.088 |
| Gender |  |  |  |  |
| Female | Ref |  | Ref |  |
| Male | -0.68 (-0.87, -0.48) | <0.001 | -0.29 (-0.47, -0.12) | 0.001 |
| Ethnicity |  |  |  |  |
| Black | Ref |  |  |  |
| Others | -0.23 (-0.76, 0.29) | 0.381 |  |  |
| Unknown | -0.40 (-0.93, 0.13) | 0.138 |  |  |
| White | -0.40 (-0.87, 0.07) | 0.097 |  |  |
| Insurance |  |  |  |  |
| Medicaid | Ref |  |  |  |
| Medicare | 0.49 (0.02, 0.95) | 0.043 |  |  |
| Others | -0.04 (-0.51, 0.42) | 0.850 |  |  |
| Marital status |  |  |  |  |
| Married | Ref |  |  |  |
| Non-married | 0.46 (0.27, -0.65) | <0.001 |  |  |
| Unknown | 0.51 (0.13, 0.88) | 0.009 |  |  |
| 24 h urine output | -0.00 (-0.00, -0.00) | <0.001 | -0.00 (-0.00, -0.00) | <0.001 |
| Alcohol abuse |  |  |  |  |
| No | Ref |  |  |  |
| Yes | 0.25 (-0.58, 1.07) | 0.558 |  |  |
| Weight | -0.00 (-0.00, 0.00) | 0.855 |  |  |
| SAPSII | 0.05 (0.04, 0.06) | <0.001 | 0.01 (0.00, 0.02) | 0.001 |
| Charlson comorbidity index | 0.41 (0.36, 0.46) | <0.001 | 0.19 (0.14-0.24) | <0.001 |
| Heart rate | 0.03 (0.02, 0.04) | <0.001 | 0.01 (0.01, 0.02) | <0.001 |
| Systolic blood pressure | -0.01 (-0.01, -0.00) | 0.037 | -0.00 (-0.01, 0.00) | 0.083 |
| Diastolic blood pressure | -0.02 (-0.03, -0.01) | <0.001 |  |  |
| CVP | 0.00 (-0.00, 0.01) | 0.213 |  |  |
| Respiratory rate | 0.05 (0.02, 0.07) | 0.001 |  |  |
| Temperature | -0.29 (-0.44, -0.14) | <0.001 |  |  |
| SPO_2_ | -0.18 (-0.22, -0.14) | <0.001 | -0.06 (-0.09, -0.02) | 0.003 |
| WBC | 0.03 (0.01, 0.04) | <0.001 |  |  |
| Platelet | -0.00 (-0.00, -0.00) | 0.003 |  |  |
| RDW | 0.35 (0.28, 0.41) | <0.001 | 0.05 (-0.01, 0.11) | 0.100 |
| Hematocrit | -0.05 (-0.06--0.03) | <0.001 |  |  |
| INR | 1.55 (1.25, 1.86) | <0.001 | 0.39 (0.12, 0.66) | 0.005 |
| BUN | 0.05 (0.04, 0.06) | <0.001 |  |  |
| Glucose | 0.00 (0.00, 0.01) | <0.001 | -0.00 (-0.00, 0.00) | 0.167 |
| Calcium | 0.14 (-0.00, 0.28) | 0.053 |  |  |
| Sodium | -0.01 (-0.04, 0.02) | 0.479 |  |  |
| Potassium | -0.03 (-0.14, 0.09) | 0.639 |  |  |
| Chloride | 0.01 (-0.01, 0.02) | 0.409 |  |  |
| Bicarbonate | -0.18 (-0.22, -0.14) | <0.001 | -0.04 (-0.08, -0.01) | 0.019 |
| Lactate | 0.43 (0.35, 0.51) | <0.001 | 0.10 (0.03, 0.18) | 0.009 |
| PH | -1.69 (-3.10, -0.29) | 0.018 |  |  |
| Paco2 | 0.00 (-0.01, 0.02) | 0.781 |  |  |
| Pao2 | -0.00 (-0.00, 0.00) | 0.064 |  |  |
| Surgery type |  |  |  |  |
| Aortic replacement | Ref |  | Ref |  |
| CABG | -1.89 (-2.37, -1.42) | <0.001 | -1.10 (-1.52, -0.68) | <0.001 |
| Combined cardiac surgery | -0.70 (-1.21, -0.18) | 0.008 | -0.57 (-1.03, -0.11) | 0.015 |
| Repair of septal defect of heart | -2.42 (-3.97, -0.87) | 0.002 | -1.30 (-2.64, 0.05) | 0.059 |
| Valvular surgery | -1.43 (-1.94, -0.92) | <0.001 | -0.76 (-1.21, -0.30) | 0.001 |
| Extracorporeal circulation |  |  |  |  |
| No | Ref |  | Ref |  |
| Yes | -0.26 (-0.44, -0.09) | 0.004 | -0.28 (-0.45, -0.10) | 0.002 |
| Ventilation |  |  |  |  |
| No | Ref |  |  |  |
| Yes | 1.17 (0.33, 2.01) | 0.006 |  |  |
| Midazolam |  |  |  |  |
| No | Ref |  | Ref |  |
| Yes | 5.56 (5.12, 6.00) | <0.001 | 4.21 (3.79, 4.62) | <0.001 |
| Propofol |  |  |  |  |
| No | Ref |  |  |  |
| Yes | 0.72 (-0.08, 1.52) | 0.078 |  |  |
| Dexmedetomidine |  |  |  |  |
| No | Ref |  | Ref |  |
| Yes | 1.33 (1.12, 1.54) | <0.001 | 0.86 (0.67, 1.04) | <0.001 |
| Antiplatelet |  |  |  |  |
| No | Ref |  | Ref |  |
| Yes | -0.64 (-2.02, 0.74) | 0.365 | 0.47 (0.27, 0.67) | <0.001 |
| Anticoagulation |  |  |  |  |
| No | Ref |  | Ref |  |
| Yes | 1.77 (1.60, 1.94) | <0.001 | 1.07 (0.91, 1.23) | <0.001 |
| EGFR | 0.00 (0.00, 0.00) | <0.001 |  |  |
| Delirium |  |  |  |  |
| No | Ref |  | Ref |  |
| Yes | 2.63 (2.32, 2.95) | <0.001 | 1.59 (1.30, 1.87) | <0.001 |
| Anemia |  |  |  |  |
| No | Ref |  |  |  |
| Yes | -0.24 (-0.53, 0.06) | 0.115 |  |  |

β: Coefficient; CI: Confidence intervals; Ref: reference; SAPSII: Simplified Acute Physiology Score II; CVP: central venous pressure; SpO2: oxygen saturation; WBC: white blood cell; RDW: red cell distribution width; INR: international normalized ratio; BUN: blood urea nitrogen; PaO_2_: partial pressure of oxygen; CABG: coronary artery bypass graft; eGFR: estimated glomerular filtration rate

Model 1: the crude model

Model 2: multivariable model after bidirectional stepwise regression

Supplementary Table 4 The screening process of the potential confounding factors associated with in-hospital mortality

|  | Model 1 |  | Model 2 |  |
| --- | --- | --- | --- | --- |
| Variables | HR (95% CI) | *P* | HR (95% CI) | *P* |
| Age | 1.01 (0.97-1.04) | 0.737 |  |  |
| Gender |  |  |  |  |
| Female | Ref |  |  |  |
| Male | 0.94 (0.47-1.87) | 0.865 |  |  |
| Ethnicity |  |  |  |  |
| Black | Ref |  |  |  |
| Others | 1.19 (0.13-10.80) | 0.874 |  |  |
| Unknown | 3.30 (0.41-26.45) | 0.261 |  |  |
| White | 1.33 (0.18-9.88) | 0.780 |  |  |
| Insurance |  |  |  |  |
| Medicaid | Ref |  |  |  |
| Medicare | 2.58 (0.34-19.50) | 0.359 |  |  |
| Others | 2.01 (0.26-15.40) | 0.500 |  |  |
| Marital status |  |  |  |  |
| Married | Ref |  |  |  |
| Non-married | 0.71 (0.32-1.57) | 0.398 |  |  |
| Unknown | 2.92 (1.23-6.94) | 0.015 |  |  |
| 24 h urine output | 1.00 (1.00-1.00) | <0.001 |  |  |
| Alcohol abuse |  |  |  |  |
| No | Ref |  |  |  |
| Yes | 0.00 (0.00-Inf) | 0.996 |  |  |
| Weight | 1.00 (1.00-1.00) | 0.914 |  |  |
| SAPSII | 1.07 (1.04-1.09) | <0.001 | 1.04 (1.02-1.07) | <0.001 |
| Charlson comorbidity index | 1.14 (0.97-1.34) | 0.100 |  |  |
| Heart rate | 1.03 (1.01-1.06) | 0.010 |  |  |
| Systolic blood pressure | 0.99 (0.97-1.01) | 0.346 |  |  |
| Diastolic blood pressure | 0.99 (0.96-1.02) | 0.558 |  |  |
| CVP | 1.01 (1.00-1.02) | <0.001 | 1.01 (1.00-1.02) | 0.047 |
| Respiratory rate | 1.11 (1.06-1.16) | <0.001 |  |  |
| Temperature | 0.62 (0.37-1.04) | 0.072 |  |  |
| SPO2 | 0.93 (0.89-0.98) | 0.003 |  |  |
| WBC | 1.01 (0.97-1.06) | 0.651 |  |  |
| Platelet | 0.99 (0.98-1.00) | 0.053 |  |  |
| RDW | 1.21 (1.04-1.42) | 0.017 |  |  |
| Hematocrit | 1.00 (0.94-1.05) | 0.894 |  |  |
| INR | 1.65 (1.35-2.02) | <0.001 |  |  |
| BUN | 1.02 (1.00-1.04) | 0.113 |  |  |
| Glucose | 1.01 (1.01-1.02) | <0.001 |  |  |
| Calcium | 1.44 (1.27-1.65) | <0.001 | 1.22 (1.04-1.43) | 0.012 |
| Sodium | 1.18 (1.07-1.31) | 0.001 |  |  |
| Potassium | 0.82 (0.53-1.25) | 0.348 |  |  |
| Chloride | 1.00 (0.95-1.05) | 0.967 |  |  |
| Bicarbonate | 0.71 (0.63-0.81) | <0.001 | 0.84 (0.74-0.96) | 0.009 |
| Lactate | 1.87 (1.67-2.11) | <0.001 | 1.34 (1.16-1.55) | <0.001 |
| PH | 0.00 (0.00-0.00) | <0.001 |  |  |
| Paco2 | 1.04 (0.99-1.08) | 0.097 |  |  |
| Pao2 | 1.00 (1.00-1.00) | 0.347 |  |  |
| Surgery type |  |  |  |  |
| Aortic replacement | Ref |  | Ref |  |
| CABG | 0.12 (0.05-0.30) | <0.001 | 0.31 (0.11-0.87) | 0.027 |
| Combined cardiac surgery | 0.31 (0.13-0.77) | 0.012 | 0.38 (0.13-1.11) | 0.077 |
| Repair of septal defect of heart | 2.88 (0.35-23.59) | 0.323 | 3.59 (0.31-41.51) | 0.305 |
| Valvular surgery | 0.18 (0.06-0.54) | 0.002 | 0.33 (0.09-1.16) | 0.083 |
| Extracorporeal circulation |  |  |  |  |
| No | Ref |  |  |  |
| Yes | 1.59 (0.80-3.17) | 0.184 |  |  |
| Ventilation |  |  |  |  |
| No | Ref |  |  |  |
| Yes | 3321992.78 (0.00-Inf) | 0.997 |  |  |
| Midazolam |  |  |  |  |
| No | Ref |  | Ref |  |
| Yes | 22.40 (10.56-47.48) | <0.001 | 7.20 (2.96-17.51) | <0.001 |
| Propofol |  |  |  |  |
| No | Ref |  |  |  |
| Yes | 0.29 (0.04-2.15) | 0.226 |  |  |
| Dexmedetomidine |  |  |  |  |
| No | Ref |  |  |  |
| Yes | 0.68 (0.31-1.50) | 0.336 |  |  |
| Antiplatelet |  |  |  |  |
| No | Ref |  | Ref |  |
| Yes | 0.04 (0.01-0.14) | <0.001 | 0.09 (0.02-0.38) | 0.001 |
| Anticoagulation |  |  |  |  |
| No | Ref |  |  |  |
| Yes | 2.09 (0.94-4.63) | 0.069 |  |  |
| Length of ICU stay | 1.00 (0.96-1.05) | 0.926 | 0.94 (0.88-1.00) | 0.060 |
| EGFR | 1.00 (1.00-1.00) | 0.051 |  |  |
| Delirium |  |  |  |  |
| No | Ref |  | Ref |  |
| Yes | 0.49 (0.16-1.49) | 0.208 | 0.31 (0.09-1.04) | 0.059 |
| Anemia |  |  |  |  |
| No | Ref |  |  |  |
| Yes | 1.00 (0.31-3.28) | 0.998 |  |  |

HR: Hazard ratio; CI: Confidence intervals; Ref: reference; SAPSII: Simplified Acute Physiology Score II; CVP: central venous pressure; SpO2: oxygen saturation; WBC: white blood cell; RDW: red cell distribution width; INR: international normalized ratio; BUN: blood urea nitrogen; PaO2: partial pressure of oxygen; CABG: coronary artery bypass graft; eGFR: estimated glomerular filtration rate

Model 1: the crude model

Model 2: multivariable model after bidirectional stepwise regression
